# Supplementary material for: Ion diffusion retarded by diverging chemical susceptibility
Source: Nat Commun. 2024 Jul 10;15:5814. doi: 10.1038/s41467-024-50213-3 (PMC11237041; doi:10.1038/s41467-024-50213-3)
Supplement: Supplementary file 1 — Supplementary Information [file 41467_2024_50213_MOESM1_ESM.pdf]

Supplementary Information

Ion Diffusion Retarded by Diverging Chemical Susceptibility

Yuhang Cai, Zhaowu Wang, Jiawei Wan, Jiachen Li, Ruihan Guo, Joel W. Ager, Ali Javey, Haimei Zheng, Jun Jiang, and Junqiao Wu

This PDF file includes:

Supplementary Figures 1-14
Supplementary Tables 1-6
Supplementary Notes 1-5

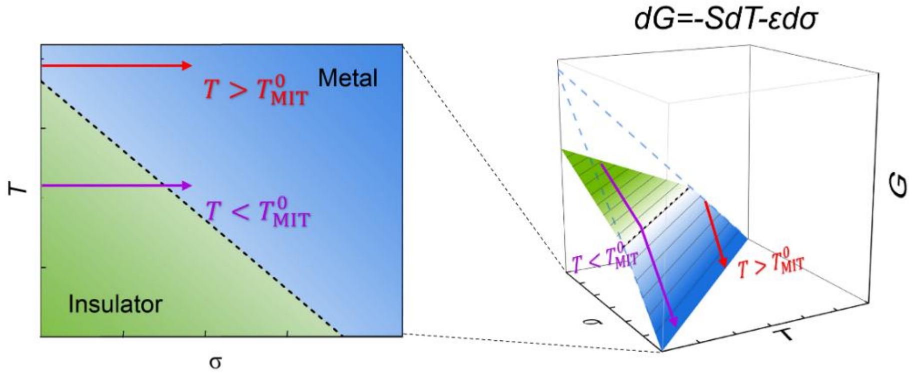

Supplementary Figure 1. Schematic of uniaxial compressive stress (along [001]$_{\text{M-phase, rutile}}$ axis)-temperature phase diagram of VO_2. Left panel shows the phase diagram in the T - σ space[1]. Right panel: Gibbs free energy of the metal (M) and insulator (I) phases of VO_2 as a function of temperature (T) and compressive stress (σ) The two arrows show the paths for compressive stress at T higher or lower than T^0_{MIT}, corresponding to the linear stress-strain relationship and super-elasticity, respectively[2].

Supplementary Note 1. MIT of VO_2 driven by temperature (T), uniaxial stress (σ), and chemical doping (x).

The differential enthalpy (H) and Gibbs free energy (G) of the can be written as:

$$\begin{cases} dH = -TdS - \varepsilon d\sigma + \gamma dx \\ dG = d(H - TS) = -SdT - \varepsilon d\sigma + \gamma dx \end{cases} \quad (1)$$

The first derivative of G versus the variables is entropy (-S), uniaxial strain (-ε), and formation energy per hydrogen atom (γ). Here γ is defined as:

$$\gamma = [H(\mathrm{H}_x\mathrm{VO}_2) - H(\mathrm{VO}_2)]/x - E(\mathrm{H}_2)/2, \quad (2)$$

where $E(\mathrm{H}_2)$ is a constant independent of the VO_2 phase. It is noted that the formation energy (γ) in Supplementary Equation (1) is primarily related to the enthalpy of the H_xVO_2 system, and is distinct from the chemical potential (μ) in typical binary systems which is continuous across a phase transition, as clarified in Supplementary Table 1.

The MIT, as a first-order phase transition, is a competition between the higher entropy of the M phase and the lower enthalpy of I phase[3]. As a result, latent heat is present at the transition point ($\Delta H = T_{\text{MIT}}\Delta S$), resulting in divergent heat capacity ($\sim dS/dT$).

With uniaxial compressive stress σ, the transition temperature (T_{MIT}) decreases following the Clausius–Clapeyron equation: $dT_{\text{MIT}}/d\sigma = \Delta\varepsilon/\Delta S$. The higher entropy of M phase mainly comes from softer phonons and the available electronic states at the Fermi level, therefore ΔS is naturally assumed to be nearly a constant[4]. The reduction of ΔH comes from the intrinsic strain $\Delta\varepsilon$ at the transition point:

$$\Delta H^0 - \Delta H^\sigma = \sigma\Delta\varepsilon = \Delta T_{\text{MIT}}\Delta S, \quad (3)$$

leading to the Clausius–Clapeyron equation, and has been experimentally demonstrated[2].

With external stress σ applied, $\sigma\Delta\varepsilon$ compensates for the reduction of latent heat; the intrinsic strain ($\Delta\varepsilon$) at the transition point results in a divergent compressibility ($\sim d\varepsilon/d\sigma$).

Similarly, with chemical doping, the transition temperature (together with ΔH) decreases based on Supplementary Equation (1). The reduction of ΔH comes from a discontinuous formation energy, marked as γ in this work,

$$\Delta H^0 - \Delta H^x = -x\Delta\gamma = \Delta T_{\mathrm{MIT}}\Delta S, \tag{4}$$

where $\Delta\gamma = \gamma_{\mathrm{M}} - \gamma_{\mathrm{I}} < 0$, representing the difference in formation energy of placing a hydrogen atom into the M-phase VO_2 and I-phase VO_2. The existence of $\Delta\gamma$ has been verified theoretically in our study (Supplementary Table 4) as well as by previous work[5,6]. With chemical doping, $-x_{\mathrm{MIT}}\Delta\gamma$ compensates for the reduction of latent heat; the energy difference of $\Delta\gamma$ at the transition point results in a divergent susceptibility ($\sim d\gamma/dx$). Therefore, T, σ, x and S, ε, γ are fundamentally analogous, as shown in the main text (Fig. 1a).

Supplementary Table 1. Difference between typical binary systems and our work.

	Binary systems	Our work
System configuration	$A_{x_A}B_{x_B}$	H_xVO_2
	Phase transition between α and β	Phase transition between M and I
	Mixing of A and B	Diffusion of H into VO_2
	$x_A + x_B = 1$, No minority	$x_H = x$ varies, H is minority, VO_2 amount is fixed
	In equilibrium	Dynamic diffusion
Key variables and equations	Gibbs free energy (G)	Enthalpy (H)
	Chemical potential (μ)	Hydrogen formation energy (γ)
	$G = x_A\mu_A + x_B\mu_B$	$H(H_xVO_2) = H(VO_2) + x\cdot\gamma$
Continuity at phase transition	$\mu_\alpha = \mu_\beta$	$\gamma_{\mathrm{M}} \neq \gamma_{\mathrm{I}}$

a

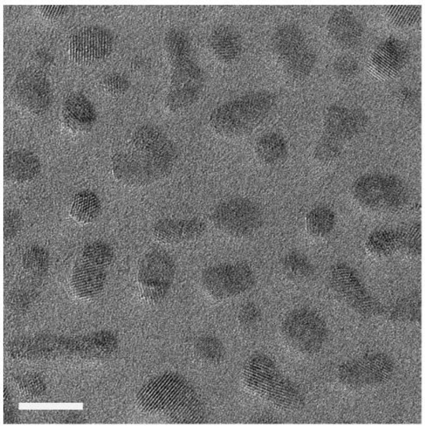

b

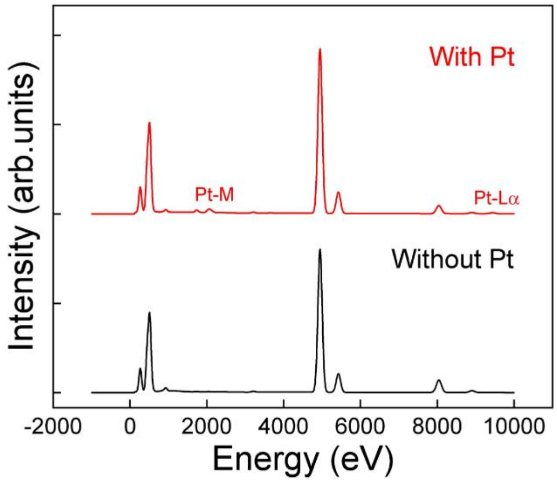

Supplementary Figure 2. Pt nano-islands as catalyst of hydrogen spillover[7,8]. a TEM image of platinum (Pt) nano-islands after 0.3-nm Pt deposition. Scale bar: 5nm. **b** Energy-dispersive X-ray spectra of a VO_2 microbeam with and without Platinum deposition.

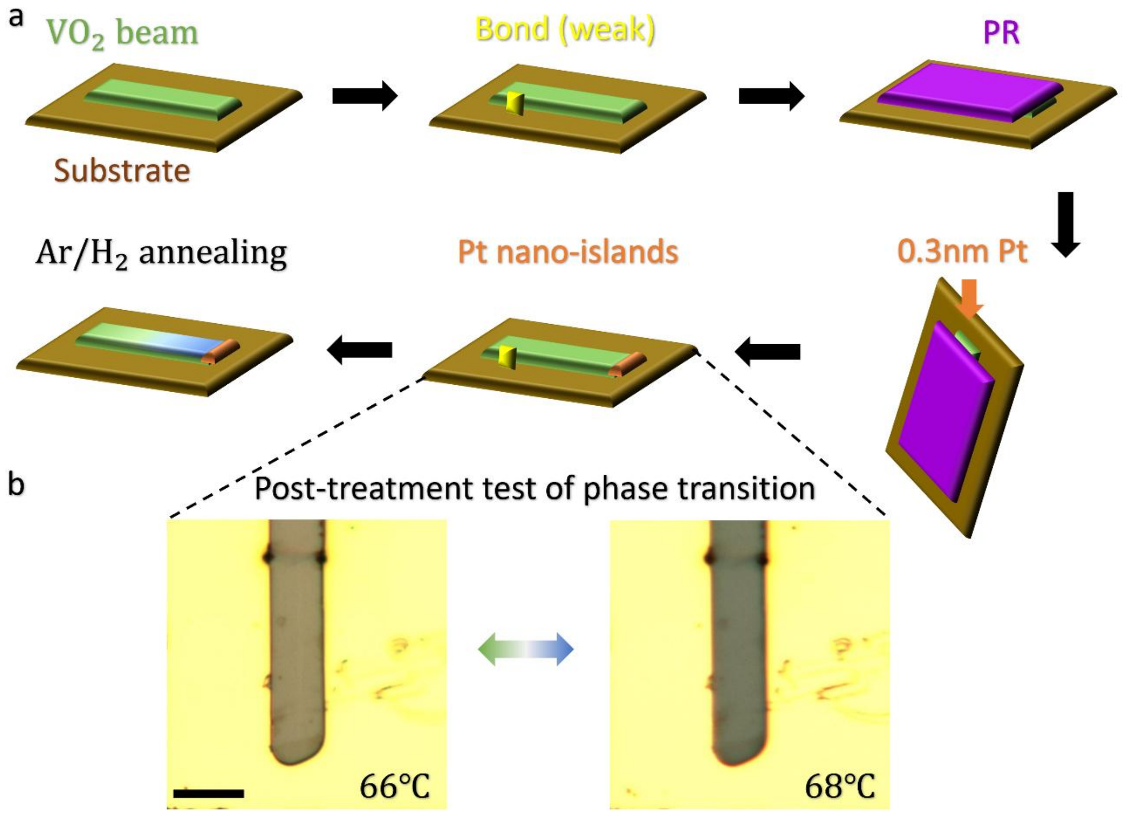

Supplementary Figure 3. Device fabrication and hydrogenation of VO_2. a As-grown single-crystalline microbeams are transferred onto Au pads, which function as bottom markers. Metal is deposited using electron beam deposition to secure the beams as a bond[9]. The bonding is weak enough to avoid any interfacial strain. In addition, the bonds are far away from the beam edge so that they don't influence the catalytic behavior at the tip area. After being secured, the microbeams are covered by photoresist with the tip area exposed for e-beam evaporation of catalyst. It is noted that part of the top surface is also exposed to catalyst evaporation, but due to the large anisotropy (Fig. 3 and Supplementary Fig. 5) of hydrogen diffusion, it won't affect the measurement of hydrogen diffusion length along $[001]_{\text{M-phase, rutile}}$ axis. **b** Sharp phase transition of VO_2 microbeams with weak bonds, which indicates the negligible interfacial strain[10]. Scale bar: 10nm.

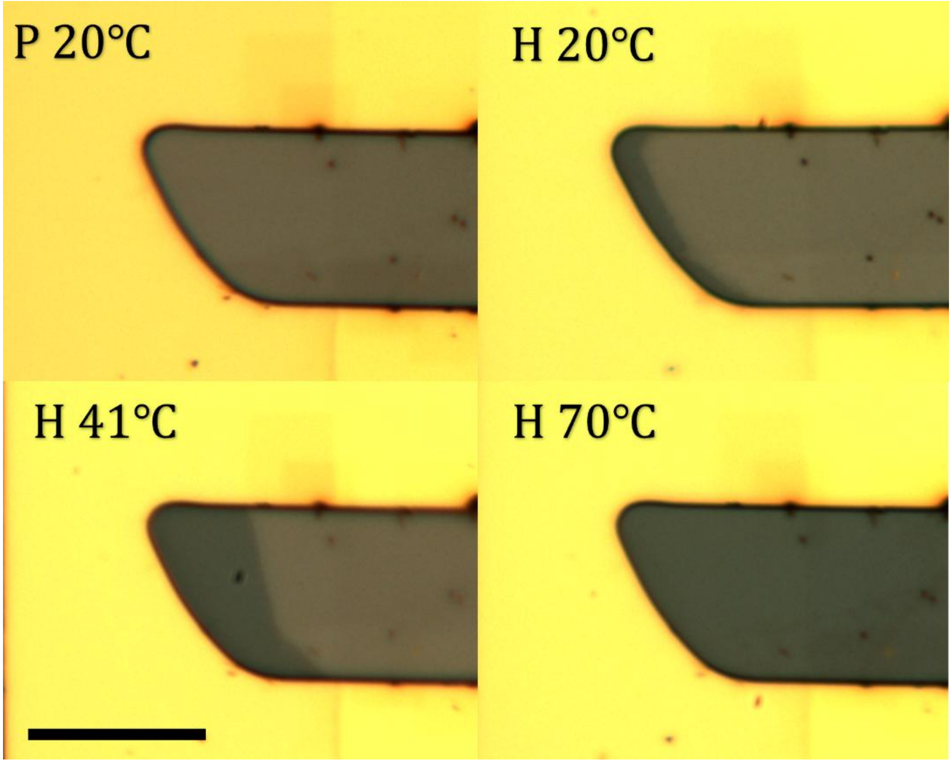

Supplementary Figure 4. Optical images of pristine (P) and hydrogenated (H) VO_2 microbeams. While T_{MIT} of hydrogen doped VO_2 decreases with increasing doping concentration (Fig. 1b), the gradually moving domain boundary demonstrates the gradient doping along the beam direction[11]. $T_{\mathrm{anneal}} = 41$°C. Scale bar: 15μm.

Supplementary Note 2. Error bar (ΔL_{M}) of the diffusion length (L_{M}) and diffusivity (D)

In Fig. 1c, $\ln D = \ln\,(L_{\mathrm{M}}^2/t)$, the error bar of $\ln D$ is determined mathematically:

$$\Delta(\ln D) = \Delta D/D = 2L_{\mathrm{M}}\Delta L_{\mathrm{M}}/Dt = 2\Delta L_{\mathrm{M}}/L_{\mathrm{M}}, \qquad (5)$$

ΔL_{M} comes from the measurement of L_{M} on multiple samples. In Fig. 2b, triangular and circular data points represent independent measurements on two microbeams. Based on the abrupt change of Raman intensity at 612cm^{-1}, L_{M} and ΔL_{M} are determined to be 5.5μm and 0.5μm, respectively. According to Supplementary Equation (5), $\Delta(\ln D) = 0.18$. In Fig. 2c, the overall error bar of D at a specific temperature (e.g. 56°C) depends on the data points with larger t (72h for 56°C, Fig. 2b). Therefore, $\Delta(\ln D) = 0.18$ at 56°C.

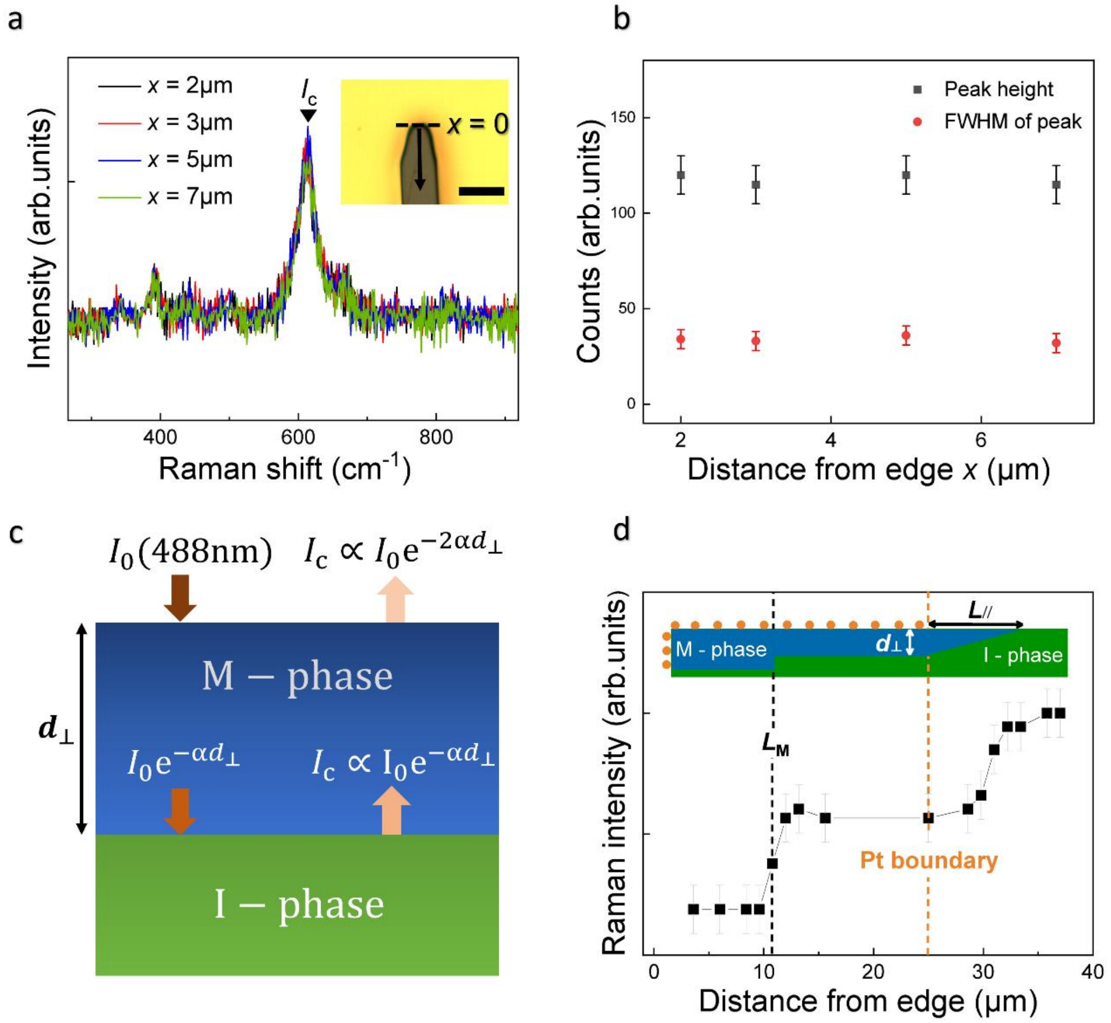

Supplementary Figure 5. Anisotropy of hydrogen diffusion in VO_2. a b Constant Raman peak intensity[12] (peak height and width) at ~612cm^{-1} (labeled as I_c) of pristine VO_2 single crystals. It's the basis of determining the diffusion depth based on Raman peak intensity. Error bar: reading error of Raman peak width and height. **c** Mechanism of determining the hydrogen diffusion depth by I_c. The wavelength of Raman laser beam is 488nm, at which the optical properties of insulating and metallic VO_2 are identical[4]. Therefore, I_c at ~612cm^{-1} (insulating VO_2) is proportional to the decayed laser intensity: $I_c \propto \exp(-2\alpha d_\perp)$, α is the absorption coefficient of VO_2 at 488nm, $d_\perp$ is the hydrogen diffusion depth. **d** Mapping of I_c along a hydrogenated VO_2 microbeam. Deposition of catalyst on both the tip area and top surface makes it possible to compare the hydrogen diffusivity along $[001]_{\text{M-phase, rutile}}$ and $[110]_{\text{M-phase, rutile}}$ (Supplementary Fig. 7). Since $d_\perp$ is ~100nm while L_M and $L_\parallel$ are ~10μm, the diffusivity along $[001]_{\text{M-phase, rutile}}$ is approximately 4 orders of magnitude larger than that of $[110]_{\text{M-phase,}}$ rutile. Hydrogen annealing temperature: 120°C. Error bar: reading error of Raman peak intensity.

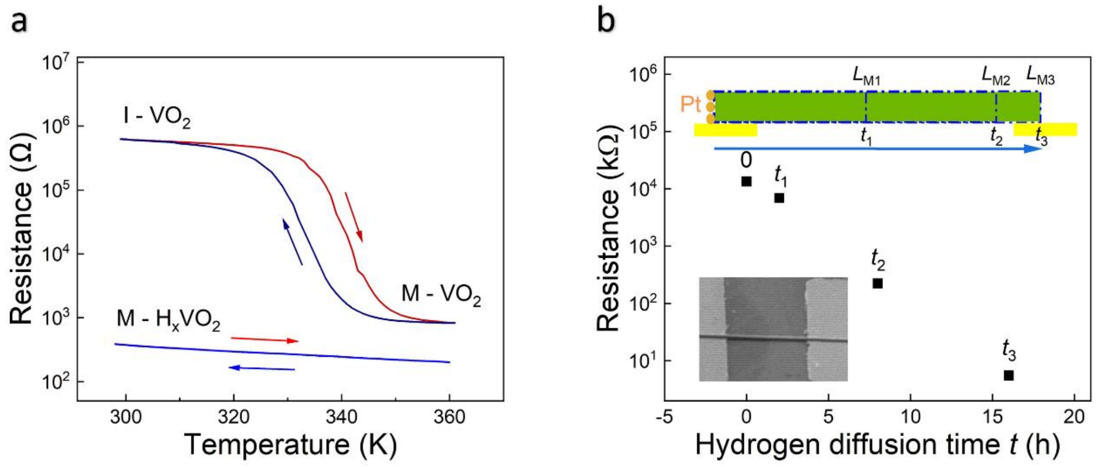

Supplementary Figure 6. stabilization of metallic phase by atomic hydrogen diffusion. a Temperature dependent resistance of pristine VO_2 and metallic hydrogenated VO_2 films. **b** Time dependent resistance of a VO_2 microbeam. Catalyst is deposited on the left tip area of the microbeam using photolithography, so hydrogen diffuses along the beam direction from the left edge (blue arrow). The M-I phase boundary (L_{M}) gradually moves towards the right-side positions labeled as L_{M1}, L_{M2}, and L_{M3} in the schematic (t_1=2h, t_2=8h, t_3=16h). The positions of the M-I phase boundary (L_{M}) are estimated based on dynamic resistance of the beam[13] (R_{t}): $L_{\mathrm{M}} = L_{\mathrm{c}}(1 - R_{\mathrm{t}}/R_{\mathrm{o}})$, where L_{c} is the channel length of the device (L_{c} =10μm). Hydrogen annealing temperature: 110°C. Inlet: SEM image of a VO_2 microbeam transferred onto metal pads.

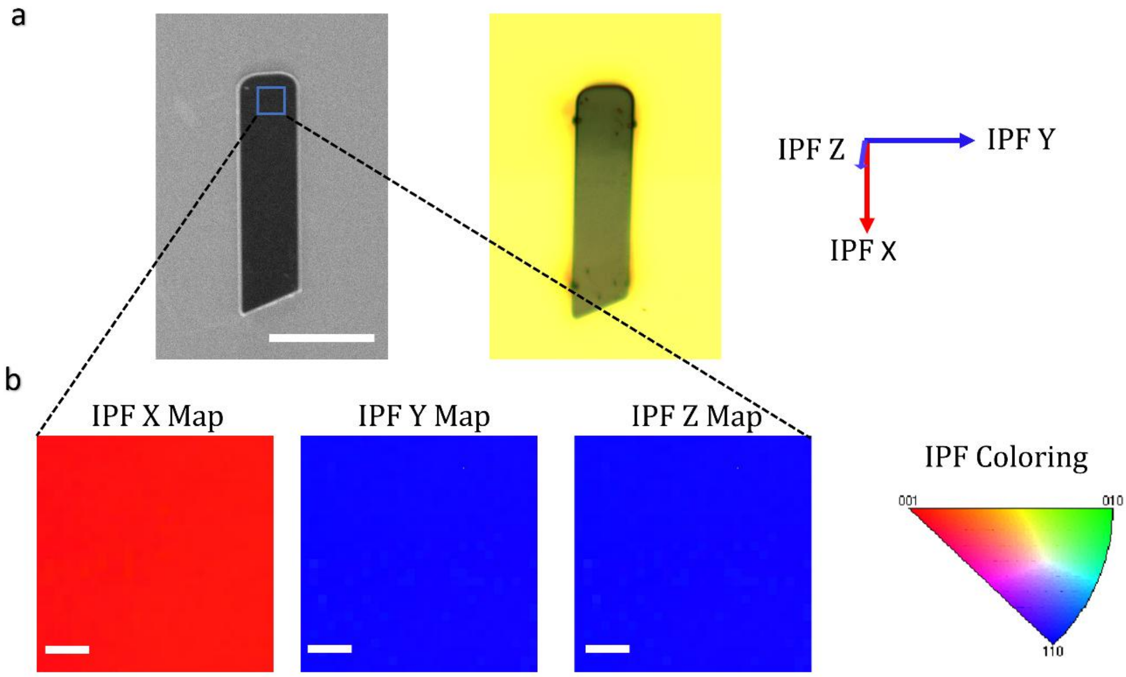

Supplementary Figure 7. Crystal orientation determined by EBSD. a SEM image and optical image of a hydrogenated microbeam. Scale bar: 20μm. The blue square (5μm x 5μm) is the area where the EBSD detector collects signal. **b** Mapping of IPF directions of the hydrogenated microbeam. The maps show the good crystallinity of the microbeam. The IPF Z direction is the normal direction of the beam's top surface ($[110]_{\text{M-phase, rutile}}$)[1] while the IPF X direction is the beam direction ($[001]_{\text{M-phase, rutile}}$), as shown in the top right panel. Scale bar: 1μm.

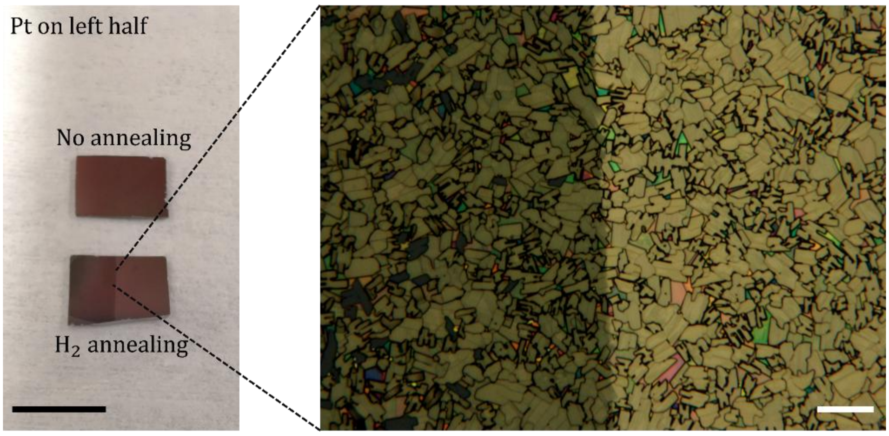

Supplementary Figure 8. Wafer-scale micro crystals for XPS measurement. To measure the chemical states (XPS) in single-crystalline VO_2 microbeams, the SiO_2/Si substrate is fully covered with VO_2 crystals. By depositing 0.3nm Pt on the left side of the substrates, a clear M-I phase boundary appears after the hydrogenation process. The Pt nano-islands don't contribute to the optical contrast just like the top substrate in the left photo. Scale bar: 1cm and 30μm, respectively.

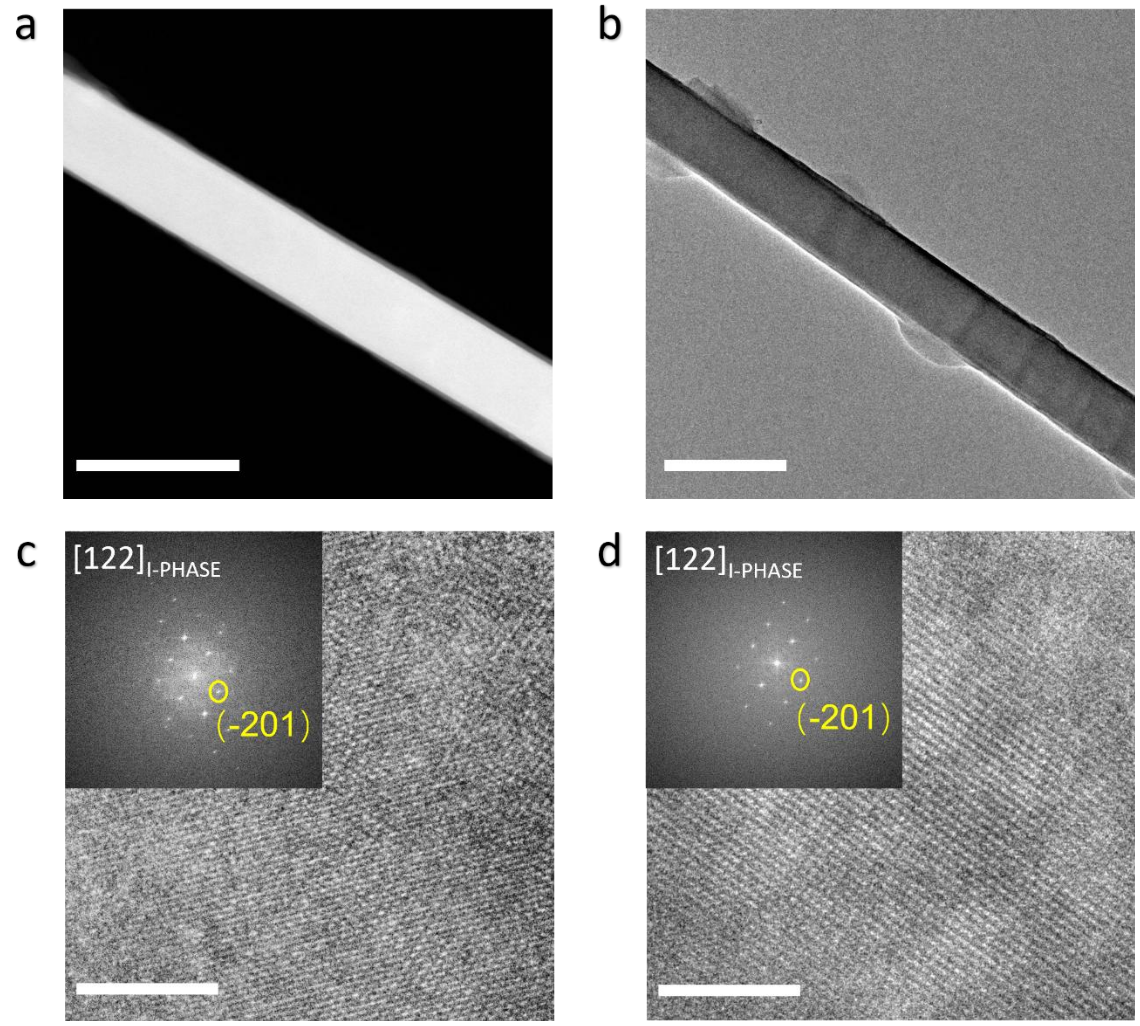

Supplementary Figure 9. TEM images of VO_2 beams. a Low-resolution HAADF image of a hydrogenated VO_2 beam. Scale bar: 500nm. **b** Low-resolution bright-field image of a hydrogenated VO_2 beam. The flocculent edge is carbon contamination, which doesn't affect the structural characterization. Scale bar: 500nm. **c d** HRTEM of multiple pristine VO_2 beams. Inlet: FFT showing the consistency of crystal orientation: axial plane of $(-201)_{\text{I-phase, monoclinic}}$ (growth axis of $[100]_{\text{I-phase,monoclinic}}$) and $[122]_{\text{I-phase, monoclinic}}$ zone axis. Scale bar: 5nm.

Supplementary Note 3. TEM characterization of monoclinic and rutile VO_2.

Previous TEM studies have shown the structural phase transition of VO_2 (monoclinic-rutile) by the absence of diffraction spots in the rutile VO_2 due to the altered symmetry[11,14]. However, the absent diffraction spot is related to specific planes[11] (e.g. $(100)_{\text{I-phase, monoclinic}}$), therefore this phenomenon doesn't always exist when the zone axis varies[15,16]. As discussed in Supplementary Fig. 7 and Supplementary Fig. 9, the zone axis of our crystals is always $[122]_{\text{I-phase, monoclinic}}$ ($[110]_{\text{M-phase, rutile}}$).

To ensure the phase transition of TEM beams, multiple single-crystalline beams are transferred for TEM characterization and electrical measurements at the same time. The electrical measurements are in the same manner as the inlet image in Supplementary Fig. 6b. 0.3nm Pt (catalyst) is deposited on all beams, followed by the identical hydrogen annealing

process. The beam resistance is measured before and after hydrogen annealing, listed in Supplementary Table 1, indicating the insulator-metal phase transition.

Supplementary Table 2. Electrical measurements of VO_2 beams as a controlled experiment for TEM.

	R before hydrogenation (MΩ)	R after hydrogenation (kΩ)
Beam 1	18.1	12.4
Beam 2	37.4	25.9

However, the monoclinic I- and rutile M-phase are not easily differentiated on the zone axis of $[122]_{\text{I-phase, monoclinic}}$ and $[110]_{\text{M-phase, rutile}}$, either in our work (Fig. 2e and 2f) or previous studies[16–18]. This is because: the inter-plane spacing of (20-1) and (0-11) planes of monoclinic VO_2 is almost the same as that of (-110) and (001) planes of rutile VO_2. Nevertheless, the angle between the two sets of planes in the monoclinic phase deviates from 90°. In addition, the carefully measured lattice parameters of pristine and hydrogenated beams confirm lattice expansion owing to hydrogen intercalation (Supplementary Table 2). The measurements are consistent although from 2 different beams before and after hydrogenation, error bars come from the variance of multiple measurements.

Supplementary Table 3. Lattice parameters of pristine and hydrogenated VO_2.

	$d[(20\text{-}1)_{\text{monoclinic}}$ or $(001)_{\text{rutile}}]$	$d[(0\text{-}11)_{\text{monoclinic}}$ or $(\text{-}110)_{\text{rutile}}]$	Dihedral angle
I, monoclinic VO_2 (theoretical)	0.287 nm	0.320 nm	89.5°
Beam 1 pristine (I, monoclinic)	0.288 ± 0.001 nm	0.320 ± 0.001 nm	89.6°±0.2°
Beam 2 pristine (I, monoclinic)	0.286 ± 0.002 nm	0.322 ± 0.002 nm	89.5°±0.3°
M, rutile VO_2 (theoretical)	0.285 nm	0.321 nm	90.0°
Beam 1 hydrogenated (M, rutile)	0.290 ± 0.001 nm	0.327 ± 0.001 nm	90.0°±0.1°
Beam 2 hydrogenated (M, rutile)	0.295 ± 0.002 nm	0.325 ± 0.002 nm	90.0°±0.2°
In-plane expansion	~4%		

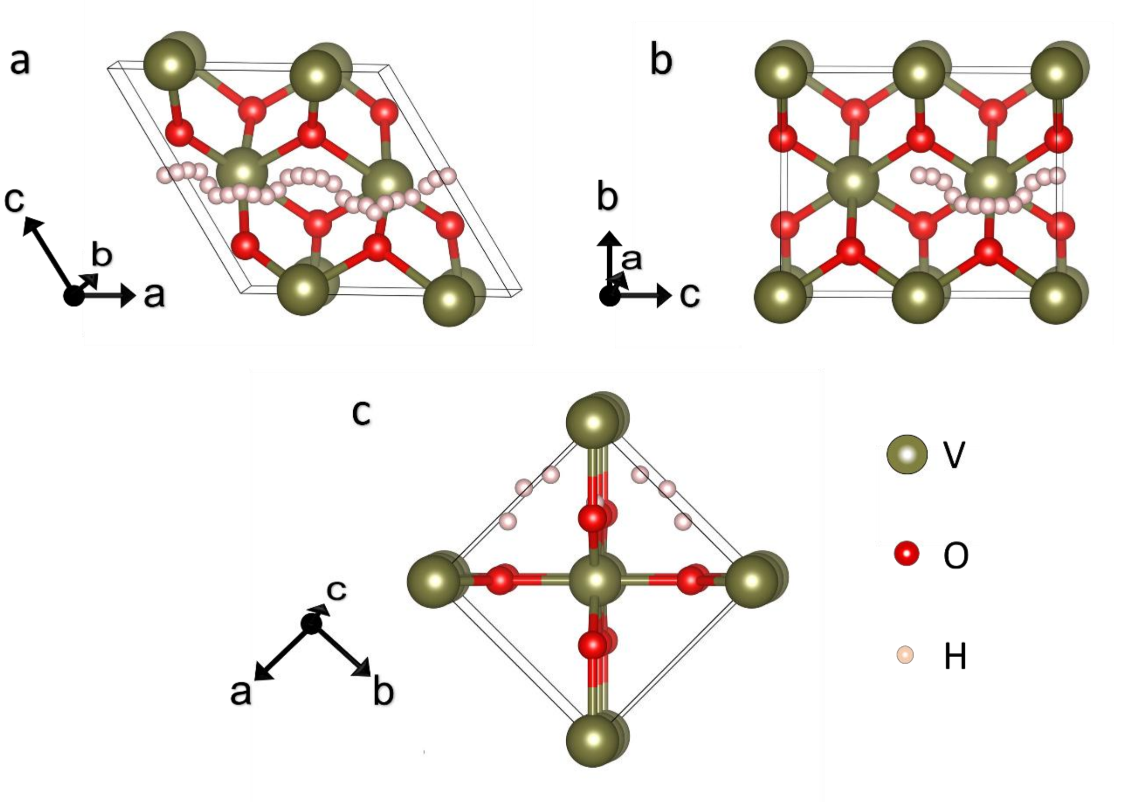

Supplementary Figure 10. Routes of hydrogen diffusion. a-c Calculated lowest-energy path for hydrogen diffusion along the $[1,0,0]_{\text{I-phase}}$, $[0,0,1]_{\text{M-phase}}$, and $[1,1,0]_{\text{M-phase}}$ directions, respectively.

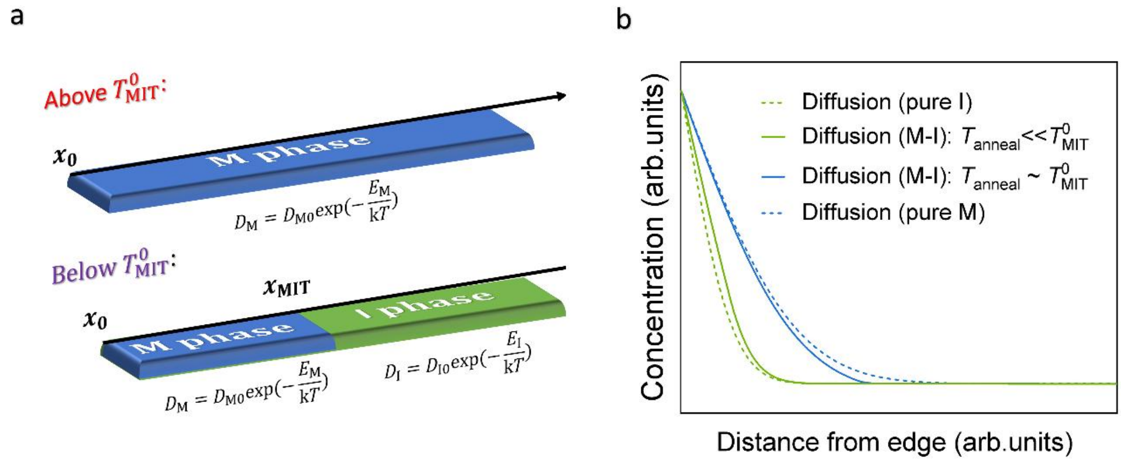

Supplementary Figure 11. Numerical simulations of hydrogen diffusion in VO_2. a Schematic of hydrogen diffusion in VO_2 below and above T^0_{MIT} for numerical simulation. While T_{anneal} is above T^0_{MIT}, the microbeam is always in M phase, the hydrogen diffusivity in M-phase VO_2 is written as $D_M = D_{M0} * \exp(-E_M/kT)$. However, there is a threshold hydrogen concentration for insulator-metal transition when $T_{anneal} < T^0_{MIT}$: $x_{MIT} = (T^0_{MIT} - T_{anneal})/(50K/\%)$[5]. Therefore, hydrogen diffusion in M-phase and I-phase VO_2 coexists with different diffusivity (D_M and D_I) and various activation barriers (E_M and E_I). By varying the pre-factors (D_{M0} and D_{I0}) in the numerical simulations, we are able to get fitting curves shown in Fig. 4a. **b** Numerical simulations of hydrogen concentration after diffusion when $D = D_M$, $D = D_I$, and D_M, D_I coexist. When $T_{anneal} \sim T^0_{MIT}$ (the temperature region slightly below T^0_{MIT}), $x_{MIT} \sim 0$ as if hydrogen diffusion was in pure M-phase VO_2, the overall effective diffusivity D is close to D_M; when $T_{anneal} \ll T^0_{MIT}$ (the temperature region far below T^0_{MIT}), $x_{MIT} \gg 0$, the overall effective diffusivity is dominated by D_I. This behavior helps explain the trend in Fig. 1c.

Supplementary Note 4. Energy penalty for hydrogen atom from M-phase to I-phase VO_2.

Considering hydrogen atoms from M-phase to I-phase VO_2, as shown in the bottom panel of Supplementary Fig. 11a, the energy penalty that diffusers need to pay is the energy difference (ΔE) between the initial and final state:

$$H_{\text{initial}} = H_{\text{M}}(\text{H}_x\text{VO}_2) + H_{\text{I}}(\text{VO}_2);\ \ H_{\text{final}} = H_{\text{M}}(\text{VO}_2) + H_{\text{I}}(\text{H}_x\text{VO}_2); \qquad (6)$$

$$\Delta E = -H_{\text{initial}} + H_{\text{final}}$$

$$= -\ [H_{\text{M}}(\text{H}_x\text{VO}_2) - H_{\text{M}}(\text{VO}_2) - xE(\text{H}_2)/2] +\ [H_{\text{I}}(\text{H}_x\text{VO}_2) - H_{\text{I}}(\text{VO}_2) - xE(\text{H}_2)/2]. \qquad (7)$$

The formation energy of x at% hydrogen is $x\gamma = H(\text{H}_x\text{VO}_2) - H(\text{VO}_2) - xE(\text{H}_2)/2$ according to Supplementary Equation (2). Therefore, the energy penalty for x mol hydrogen atoms $\Delta E = -\ x\gamma_M + x\gamma_I = -\ x\Delta\gamma$. As a result, the additional energy barrier for hydrogen diffusion is -$\Delta\gamma$/atom.

In first-principles calculations, the calculated formation energy of hydrogen is the average energy cost of adding x at% hydrogen atoms into VO_2. Therefore, the calculated formation energy varies when hydrogen concentration varies, either in our work (Supplementary Table 4) or previous reports[5]. While the formation energy is calculated, the final H_xVO_2 lattice is expanded. The internal (local) stress has already been included in the energy term of $H(\text{H}_x\text{VO}_2)$. Supercells of $HV_{32}O_{64}$ are used to find reliable formation energies of hydrogen in VO_2 because the hydrogen concentration in the supercell (3%) is on the order that we are interested in (same order as x_{MIT}). Meanwhile, to find the minimum energy paths and the transition states, Climbing Image Nudged Elastic Band (CI-NEB) method[19] is used. However, the applicability of the method to large systems (e.g. $HV_{32}O_{64}$) is limited[20], as a result, smaller supercells (HV_4O_8) are built to find the minimum energy paths (Fig. 3c to e)[21].

Supplementary Table 4. Calculated formation energy of hydrogen in VO_2.

	Enthalpy (H) (eV)	Formation energy of hydrogen (γ) (eV)
Monoclinic $V_{32}O_{64}$	-706.725	-0.118
Monoclinic $HV_{32}O_{64}$	-710.227	
Rutile $V_{32}O_{64}$	-702.352	-1.084
Rutile $HV_{32}O_{64}$	-706.820	

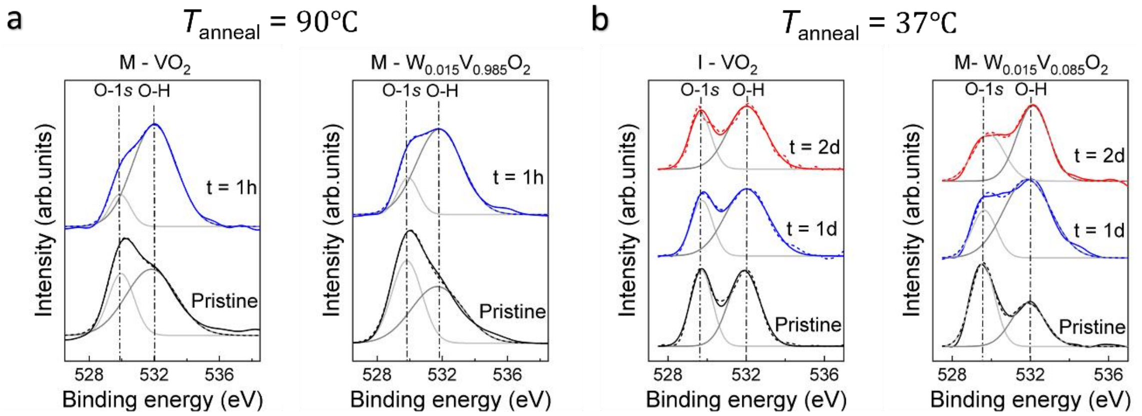

Supplementary Figure 12. Comparison of hydrogen diffusion in VO_2 and WVO_2. a XPS O-1*s* core level spectra and peak fitting of VO_2 and $W_{0.015}V_{0.985}O_2$ evolving as a function of hydrogenation hours at 90°C, showing a similar speed of hydrogen diffusion in both M-phase VO_2 and W-doped VO_2, excluding the influence of W dopants. O-H peak area (A_{OH}): ΔA_{OH} (0 to 1h) is 877 and 1031 for VO_2 and W-doped VO_2, respectively. **b** XPS O-1*s* core level spectra and peak fitting of VO_2 and $W_{0.015}V_{0.985}O_2$ evolving as a function of hydrogenation hours at 37°C, showing a much weak hydrogen diffusion in the I phase because it requires to cross M-I domain walls. O-H peak area (A_{OH}): ΔA_{OH} (0 to 2d) is 60 and 1099 for VO_2 and W-doped VO_2, respectively. Note: the absolute value of peak area varies in each measurement, the trend is more meaningful.

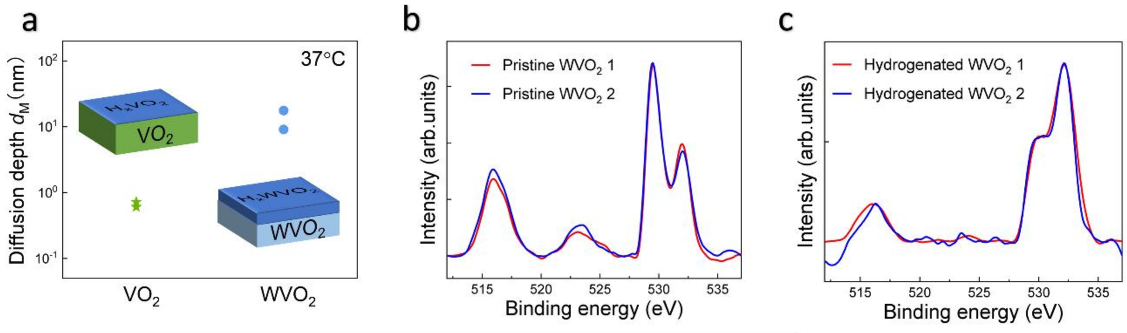

Supplementary Figure 13. Rapid hydrogen diffusion in metallic VO_2. a Estimated depth (d_{M}) of hydrogen diffusion in insulating VO_2 and metallic $W_{0.015}V_{0.985}O_2$ films (at 37°C) by the conductivity before and after hydrogenation. The conductivity is listed in Supplementary Table 5. **b c** Repeatable O-1*s* and V-2*p* core level spectra before and after hydrogenation, showing the reliability of qualitative analysis based on the O-H peak intensity at ~532.0eV in Fig. 4b and c.

Supplementary Note 5. Estimation of diffusion depth by conductivity.

Since W doping decreases T_{MIT} of VO_2 by -25K/%, T_{MIT} of $W_{0.015}V_{0.985}O_2$ (WVO_2) is around 30°C, making it possible to compare the hydrogen diffusivity in insulating and metallic VO_2 at 37°C. First, we place the films without catalyst into the tube furnace, the conductivity doesn't change much under the hydrogen environment, this is a controlled experiment to show that significant change of electrical properties comes from hydrogen intercalation.

Second, we cut all VO_2 and WVO_2 films into 2 pieces, the 1st piece is fully hydrogenated to

metallic phase, followed by the conductivity measurement of the pristine and hydrogenated pieces at room temperature, denoted as C_{P} and C_{H}, then the 2nd piece is annealed at 37°C under the forming gas containing 10% H_2. The conductivity of the 2nd, partially hydrogenated piece (schematic in Supplementary Fig. 11a) is measured at room temperature again, marked as C. Regarding the films as parallel M-phase and I-phase resistors (Supplementary Fig. 13a), the depth of M-phase (d_{M}) is:

$$d_{\mathrm{M}} = d_0 \frac{C - C_{\mathrm{P}}}{C_{\mathrm{H}} - C_{\mathrm{P}}} \tag{8}$$

where d_0 is the total thickness of the film ($d_0 = 100\mathrm{nm}$). This depth (d_{M}) is not comparable to the diffusion length (L_{M}) in the main experiment because the film is poly-crystalline and the diffusion direction is different from $[001]_{\text{M-phase, rutile}}$.[22] However, the comparison between hydrogen diffusivity in M-phase and I-phase VO_2 films is reasonable because the difference of lattice constant between $W_{0.015}V_{0.985}O_2$ and VO_2 is negligible (~1%)[23].

According to Supplementary Fig. 13a, the hydrogen diffusion depth in insulator VO_2 [d_{M}(H_xVO_2)] is negligible, consistent with the XPS results in Fig. 4b. What's more, the diffusion depth in WVO_2 [d_{M}(H_xWVO_2)] is ~1-2 orders larger than d_{M}(H_xVO_2). According to Fick's law, hydrogen diffusivity in WVO_2 is ~2-3 orders of magnitude larger at 37°C, depicted as the dashed arrow in Fig. 4a. This is a semi-quantitative comparison with a relatively large error bar because there are approximations in the analysis. For example, x_{MIT} at room temperature (where we perform all measurements) is not the same for VO_2 and WVO_2 (lower x_{MIT} for WVO_2), therefore d_{M} (H_xWVO_2) is overestimated, but within an order of magnitude.

Supplementary Table 5. Conductivity of pristine and annealed (37°C) VO_2 and WVO_2 thin films.

	C_{P}(S/m)	C_{H}(S/m)	C(S/m)
VO_2 sample 1	2.00E1	2.00E4	1.41E2
VO_2 sample 2	1.95E1	2.00E4	1.63E2
$W_{0.015}V_{0.985}O_2$ sample 1	2.02E2	1.64E4	4.54E3
$W_{0.015}V_{0.985}O_2$ sample 2	1.92E2	1.64E4	3.22E3

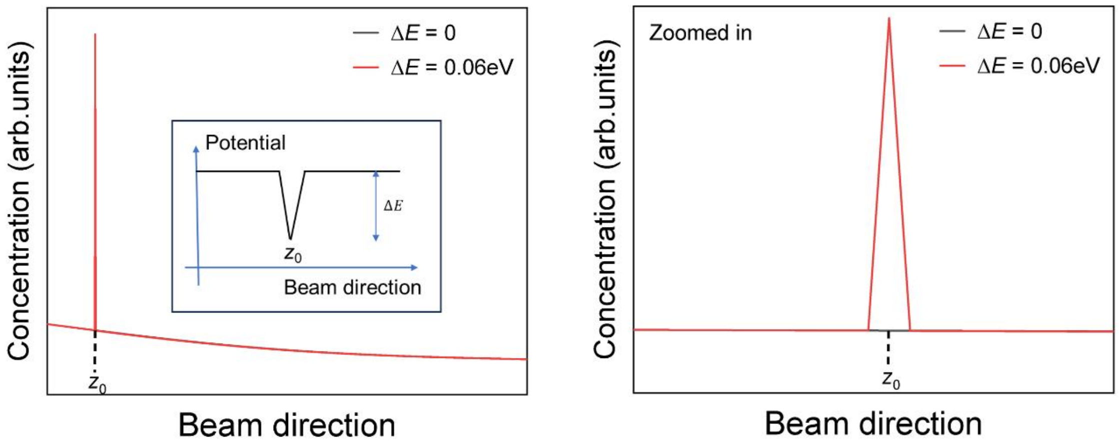

Supplementary Figure 14. Monte-Carlo simulation of particles' diffusion with particles piled up on a domain boundary. The vertical axis is hydrogen concentration in arb. units, horizontal axis is distance along the diffusion direction. The localized hydrogen concentration pile-up is found to be related to the potential dip as $x = x_0\exp(-\Delta E/kT)$, consistent with Boltzmann distribution. However, the existence of the dip and pile-up at the domain wall does not affect the overall diffusion of ions: curves with different ΔE values collapse onto each other in regions outside the dip region.

Supplementary Table 6. Additional diffusion barrier of dopants if triggering the phase transition of host materials.

	ΔS (J/mol*K)	dT_c/dx (K)	Estimated $\lvert\Delta\mu\rvert$ (eV/atom)
Li in I-VO_2 (I - M)	12.5	-44/1%	0.57
O^{2-} in O-$LaMnO_3$ (O - R)	4.6	-80/1%	0.38
C in bcc-Fe (bcc - fcc)	0.7	-50/1%	0.04
Al in Ti (bcc - hcp)	-3.7	12/1%	0.04
Fe in FM-Fe (order - disorder)			0.4

Supplementary References

1. Cao, J. *et al.* Extended Mapping and Exploration of the Vanadium Dioxide Stress-Temperature Phase Diagram. *Nano Lett.* **10**, 2667–2673 (2010).
2. Fan, W. *et al.* Superelastic metal-insulator phase transition in single-crystal VO_2 nanobeams. *Phys. Rev. B* **80**, 241105 (2009).
3. Budai, J. D. *et al.* Metallization of vanadium dioxide driven by large phonon entropy. *Nature* **515**, 535–539 (2014).
4. Liu, K., Lee, S., Yang, S., Delaire, O. & Wu, J. Recent progresses on physics and applications of vanadium dioxide. *Materials Today* **21**, 875–896 (2018).
5. Cui, Y., Shi, S., Chen, L., Luo, H. & Gao, Y. Hydrogen-doping induced reduction in the phase transition temperature of VO_2: a first-principles study. *Phys. Chem. Chem. Phys.* **17**, 20998–21004 (2015).
6. Zhang, J., He, H., Xie, Y. & Pan, B. Theoretical study on the tungsten-induced reduction of transition temperature and the degradation of optical properties for VO_2. *The Journal of Chemical Physics* **138**, 114705 (2013).
7. Yoon, H. *et al.* Reversible phase modulation and hydrogen storage in multivalent VO_2 epitaxial thin films. *Nature Mater* **15**, 1113–1119 (2016).
8. Baik, J. M. *et al.* Pd-Sensitized Single Vanadium Oxide Nanowires: Highly Responsive Hydrogen Sensing Based on the Metal−Insulator Transition. *Nano Lett.* **9**, 3980–3984 (2009).
9. Mølhave, K. *et al.* Solid Gold Nanostructures Fabricated by Electron Beam Deposition. *Nano Lett.* **3**, 1499–1503 (2003).
10. Wu, J. *et al.* Strain-Induced Self Organization of Metal−Insulator Domains in Single-Crystalline VO_2 Nanobeams. *Nano Lett.* **6**, 2313–2317 (2006).
11. Lee, S. *et al.* Axially Engineered Metal–Insulator Phase Transition by Graded Doping VO_2 Nanowires. *J. Am. Chem. Soc.* **135**, 4850–4855 (2013).
12. Zhang, Y.-Q. *et al.* Achieving room-temperature M2-phase VO_2 nanowires for superior thermal actuation. *Nano Res.* **14**, 4146–4153 (2021).
13. Wei, J., Ji, H., Guo, W., Nevidomskyy, A. H. & Natelson, D. Hydrogen stabilization of metallic vanadium dioxide in single-crystal nanobeams. *Nature Nanotech* **7**, 357–362 (2012).
14. Lee, D. *et al.* Isostructural metal-insulator transition in VO_2. *Science* **362**, 1037–1040 (2018).
15. Chen, L. *et al.* Synthesis of Thermochromic W-Doped VO_2 (M/R) Nanopowders by a Simple Solution-Based Process. *Journal of Nanomaterials* **2012**, e491051 (2012).
16. Lahneman, D. J. *et al.* Insulator-to-metal transition in ultrathin rutile VO_2/TiO_2(001). *npj Quantum Mater.* **7**, 1–8 (2022).
17. Cheng, C., Liu, K., Xiang, B., Suh, J. & Wu, J. Ultra-long, free-standing, single-crystalline vanadium dioxide micro/nanowires grown by simple thermal evaporation. *Applied Physics Letters* **100**, 103111 (2012).
18. Kang, H. *et al.* Surface hydrogeneration of vanadium dioxide nanobeam to manipulate insulator-to-metal transition using hydrogen plasma. *Journal of Asian Ceramic Societies* **9**, 1310–1319 (2021).
19. Sheppard, D., Xiao, P., Chemelewski, W., Johnson, D. D. & Henkelman, G. A generalized

solid-state nudged elastic band method. *The Journal of Chemical Physics* **136**, 074103 (2012).

20. Koistinen, O.-P., Ásgeirsson, V., Vehtari, A. & Jónsson, H. Nudged Elastic Band Calculations Accelerated with Gaussian Process Regression Based on Inverse Interatomic Distances. *J. Chem. Theory Comput.* **15**, 6738–6751 (2019).
21. Fan, L. *et al.* A Facile Strategy to Realize Rapid and Heavily Hydrogen-Doped VO_2 and Study of Hydrogen Ion Diffusion Behavior. *J. Phys. Chem. C* **126**, 5004–5013 (2022).
22. Fu, D. *et al.* Comprehensive study of the metal-insulator transition in pulsed laser deposited epitaxial VO_2 thin films. *Journal of Applied Physics* **113**, 043707 (2013).
23. Choi, S., Ahn, G., Moon, S. J. & Lee, S. Tunable resistivity of correlated VO_2(A) and VO_2(B) via tungsten doping. *Sci Rep* **10**, 9721 (2020).
